# Determinants of maternal length of stay following childbirth in a rural health facility in Eritrea

**DOI:** 10.1186/s12884-023-05931-9

**Published:** 2023-08-25

**Authors:** Ghirmay Ghebrekidan Ghebremeskel, Meron Tesfay Kahsay, Mengisteab Embaye Gulbet, Awet Ghebreberhan Mehretab

**Affiliations:** 1 Northern Red Sea branch of Ministry of the Health, Nakfa Hospital, Nakfa, Eritrea; 2 B. Sc. Clinical laboratory science, Nakfa Hospital,, Northern Red Sea branch of the Ministry of Health, Nakfa, Eritrea; 3 Adi Quala Hospital, Southern Branch of the Ministry of Health, Adi Quala, Eritrea

**Keywords:** Determinants, LOS, Maternal, Vaginal delivery, Cesarean delivery, Eritrea, Africa

## Abstract

**Background:**

The days following childbirth are a critical phase in the lives of mothers and newborns. Postpartum length of stay is a critical indicator of the efficiency of health care delivery. This study aims to explore maternal length of hospital stay (LOS) following childbirth and associated factors in a rural health facility in Eritrea.

**Methodology:**

A retrospective study of all mothers who delivered at Nakfa Hospital between 2020 and 2022 was conducted. Sociodemographic, past obstetric, and neonatal factors associated with postpartum LOS were evaluated for both vaginal delivery (VD) and cesarean delivery (CD). The determinants of LOS following VD were explored using negative binomial regression.

**Results:**

A total of 2025 mothers [1975 (97.5%) VD and 50 (2.5%) CD] were included in the study. The median LOS following childbirth was 1 (IQR: 0–1) day for VD and 6 (IQR: 4–8) days for CD. A substantial proportion of mothers were found to have inadequate stays following VD [29% (95% CI: 27–31)], whereas 68% (95% CI: 54-81%) stayed for > 4 days following CD. In this study, VD that were attended by physicians had no inadequate stay, whereas 27.4% of deliveries attended by midwives and 31.3% by associate nurses resulted in inadequate stay (*P*-value < 0.001). Determinants of LOS following VD were: the presence of maternal complications (IRR = 2.8, 95% CI: 1.6-5, *p*-value < 0.001), delivery years 2020 and 2021 (IRR = 1.5, 95% CI: 1.2–1.8, *p*-value < 0.001 and IRR = 1.4, 95% CI: 1.2–1.7, *p*-value < 0.001, respectively), and delivery hour interval 23:00–7:00 (IRR = 0.8, 95% CI: 0.7–0.9, *p*-value = 0.03).

**Conclusion:**

A substantial proportion of mothers stay too short post-VD to allow adequate postnatal care, which can have untoward consequences for maternal and child health. Going forward, improved coverage of antenatal care for early diagnosis of maternal complications in pregnancy as well as assessing the level of knowledge and provisions of training and refresher courses for birth attendants should be worked upon. In addition, efforts to conduct studies that explore maternal and health care provider perspectives on LOS should be emphasized.

## Background

Maternal length of hospital stay (LOS) has become a critical indicator of the efficiency of health care delivery; understanding factors associated with it could provide information helpful in the reduction of health care costs, improvement in the delivery of obstetric care, and containment of untoward events associated with comorbidities and complications requiring readmission [1, 2]. Globally, the magnitude of maternal and child morbidity and mortality remains enormous despite being a public health priority [3, 4]. In most settings, the magnitude of death and substantial morbidity is highest during the intrapartum and early postpartum periods, with 45-50% of maternal deaths and 24-45% of neonatal deaths occurring within this phase [5, 6]. Global health stakeholders continue to undertake efforts toward prioritizing maternal and child health. Indeed, one of the targets in the 2030 Agenda for Sustainable Development, adopted by the UN General Assembly in September 2015, is dedicated to improving maternal and child health [7].

In developing countries, a significant proportion of mothers die because it is a challenge for the existing health system to keep them in health facilities for 24 h after birth [8]. Remarkably, Sub-Saharan Africa contributes to more than half of all maternal and newborn deaths in low-income settings [6]. Generally, LOS tends to be affected by mode of delivery, with mothers delivering via cesarean section staying longer in health facilities as compared to VDs. Women who have caesarean deliveries, irrespective of demographic and obstetric characteristics, have a higher risk of maternal morbidity compared to VDs, and this could increase the duration of hospitalization [9]. WHO recommends that all women who gave birth through VD should remain admitted to hospitals or health facilities for a minimum of 24 h postpartum for observation [10]. This recommendation is to provide ample time for mothers and newborns, especially in low-income countries, to be appropriately monitored by skilled birth attendants if a serious postpartum complication arises. The American College of Obstetricians and Gynecologists (ACOG) also recommends that healthy women with uncomplicated VDs may be discharged from the hospital between 24 and 48 h after birth if they and their neonates meet specific criteria. These criteria may include stable vital signs, adequate pain control, the ability to eat and drink, uncomplicated infant feeding, and the absence of any maternal or newborn complications [11].

Despite the efforts to standardize maternal care and ensure adequate hospital stays, LOS remains low in SSA. For example, in a study in Ethiopia, the mean duration of postpartum stay for mothers in health facilities was 21.96 (95% CI: 19.97–23.94) hours, while only 34.8% of women remained in health institutions for ≥ 24 h after delivery [12]. Similarly, in their analysis of data from three Sub-Saharan African countries, Fatimah I. Tsiga-Ahmed and colleagues noted that a significant proportion of mothers remain in health facilities for less than one day following childbirth (35.1%, 18.5%, and 10.1%, respectively, in Ghana, Estwatini, and Malawi) [9].

Nevertheless, experts in this area agree that not only inadequate but also prolonged stays can have untoward consequences for maternal and child health. Indeed, both long lengths of stay and premature discharge lead to higher rates of re-admission [13] and therefore higher financial costs to families and health systems [14, 15]. In particular; Prolonged LOS has been implicated with a substantial burden on maternal and neonatal health, for example, exposure to adverse facility environments with increased risk of nosocomial infections, sleep disturbance, and higher cost [16, 17]. Interestingly, longer stays also negatively affect neonatal health, with such mothers having inappropriate breastfeeding practices [16, 18]. At the other end of the spectrum, an inadequate stay can leave insufficient time to detect, diagnose, or treat complications, which can in turn increase morbidity and mortality [19, 20]. Evidence from existing literature points out multiple factors, such as socioeconomic, demographic, and obstetric factors, in addition to neonatal factors, as determinants of maternal LOS [21, 22].

The importance of hospital-based data is therefore paramount to improving maternal care and outcomes. However, in many developing countries, health service data on postpartum morbidity remains limited [23], and information on the impact of medical and obstetrical conditions associated with pregnancies is scarce or lacking [24]. Furthermore, data from hospital-based studies is hard to interpret because of the small proportion of women who have access to supervised deliveries and medical care [23]. In Eritrea, hospital-based data regarding maternal morbidity and LOS is lacking. The existing evidence gap creates a strong demand for updated data on a range of factors associated with maternal length of hospital stay. This study was designed to explore the magnitude of prolonged and inadequate postpartum LOS in vaginal and CDs in a rural health facility in Eritrea. Additionally, the study evaluates factors associated with maternal postpartum LOS following vaginal birth and cesarean delivery.

For this study purpose, Inadequate stay following a VD was defined as < 24 h based on the ACOG recommendation for VDs, whereas prolonged stay was defined as longer than 48 h [11]. Meanwhile, prolonged postpartum stay following cesarean delivery was defined as LOS > 4 days, while LOS < 4 days was defined as inadequate stay [24, 25].

## Methodology

### Study design and setting

**Fig. 1:**
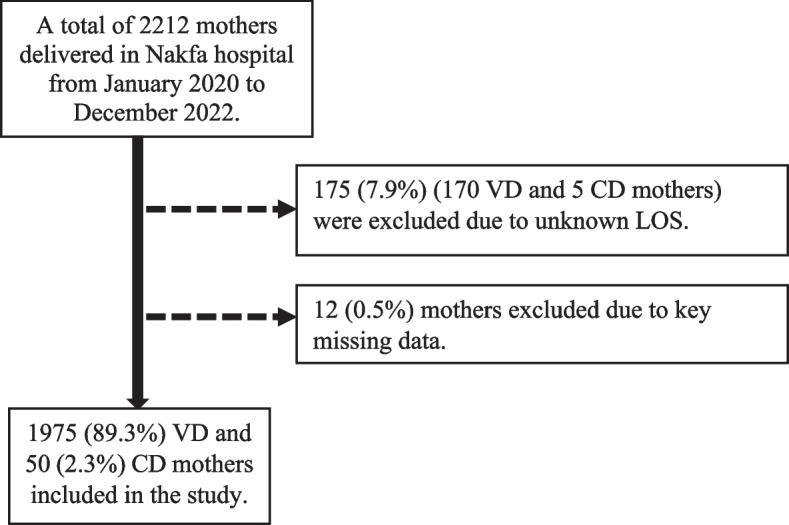
Flow chart for study participant recruitment

A retrospective cohort study was conducted to evaluate maternal and neonatal factors associated with the length of hospital stay in mothers admitted to the maternity care unit of Nakfa Hospital for delivery, from January 2020 to December 2022. Nakfa Hospital is located in Nakfa district, in the northern Red Sea zone of Eritrea. There are three health stations in Nakfa district. The maternity care unit (MCU) of Nakfa Hospital provides services to mothers from all over the district, In addition, the hospital serves as a referral institution for other health facilities in the district. The hospital also provides operative cesarean delivery (CD) services for indicated cases. In the study period, a total of 2212 mothers delivered babies at Nakfa Hospital from January 2020 to December 2022. Out of which 7.9% (175/2212) mothers were excluded due to unknown length of stay and a further 0.5% (12/2212) mothers were excluded due to key missing data (5 cases had incomplete sociodemographic characteristics and missing obstetric data, while 7 had incomplete neonatal characteristics and missing obstetric data). Finally, a total of 2025 mothers [1975 (89%) VD and 50 (2.2%) CD] were included in the study. (See Fig. 1 for details.)

### Data collection and approach

Data for this study was extracted from the maternity ward delivery register. The registry has two components: The first section focuses on maternal and labor characteristics, while the second section addresses neonatal characteristics. The following variables were recorded in the registry: age, address, gravidity, parity, abortion history, fetal presentation, mode of fetal delivery, maternal complication, admission and discharge date, delivery time, gender of the neonate, birth weight, 5-minute Apgar score, level of birth attendant, and maternal card number. Data collection was undertaken by trained health professionals and closely monitored by the principal investigators. Data from each mother’s clinical card is transferred to the respective ward’s registry at the time of discharge. The data was double-entered directly into a Microsoft Excel worksheet. The retrieved data was then de-identified, vigorously reviewed, and exported to SPSS dataset.

### Study variables

The dependent (outcome) variable of this study was the length of stay in the MCU of Hospital Nakfa after childbirth. LOS was calculated by subtracting the date of delivery from the discharge date. The independent variables were socio-demographic related factors (maternal age and distance of home from the health facility), obstetric-related factors such as gravidity, parity, abortion history, fetal presentation, mode of delivery, maternal complications, admission and discharge date, delivery time, gender of the neonate, birth weight, Apgar score, in addition to the level of birth attendant.

### Operational definition

Inadequate stay following VD was defined as < 24 h based on the ACOG recommendation for uncomplicated VDs, whereas prolonged stay was defined as longer than 48 h [12].

Prolonged postpartum stay following cesarean delivery was defined as LOS > 4 days, while LOS < 4 days was defined as inadequate stay [24, 25].

### Data analysis

All analyses were conducted using SPSS version 26 and Stata version 12.0 (Stata Corporation, College Station, TX). Descriptive statistics for categorical variables were analyzed using chi-square (χ2) or Fisher’s exact test and summarized using counts (frequency), proportions (percentages), and medians (interquartile range (IQR)). Descriptive analyses were stratified by LOS in all key variables at baseline using Pearson’s Chi-square test for categorical variables, the Mann-Whitney U test, and the Kruskal Wallis test for continuous data. Normality tests were performed before running any statistical computations. The negative binomial regression model was used to examine the factors associated with the length of stay, adjusting for other independent variables. Gravidity and Parity were highly correlated (Spearman correlation coefficient = 0.87), therefore, only parity was included in the final model. An incidence rate ratio (IRR) with a 95% confidence interval (CI) was utilized to express the magnitude of the association between the independent and dependent variables. *P* values less than 0.05 was considered significant.

## Results

**Fig. 2:**
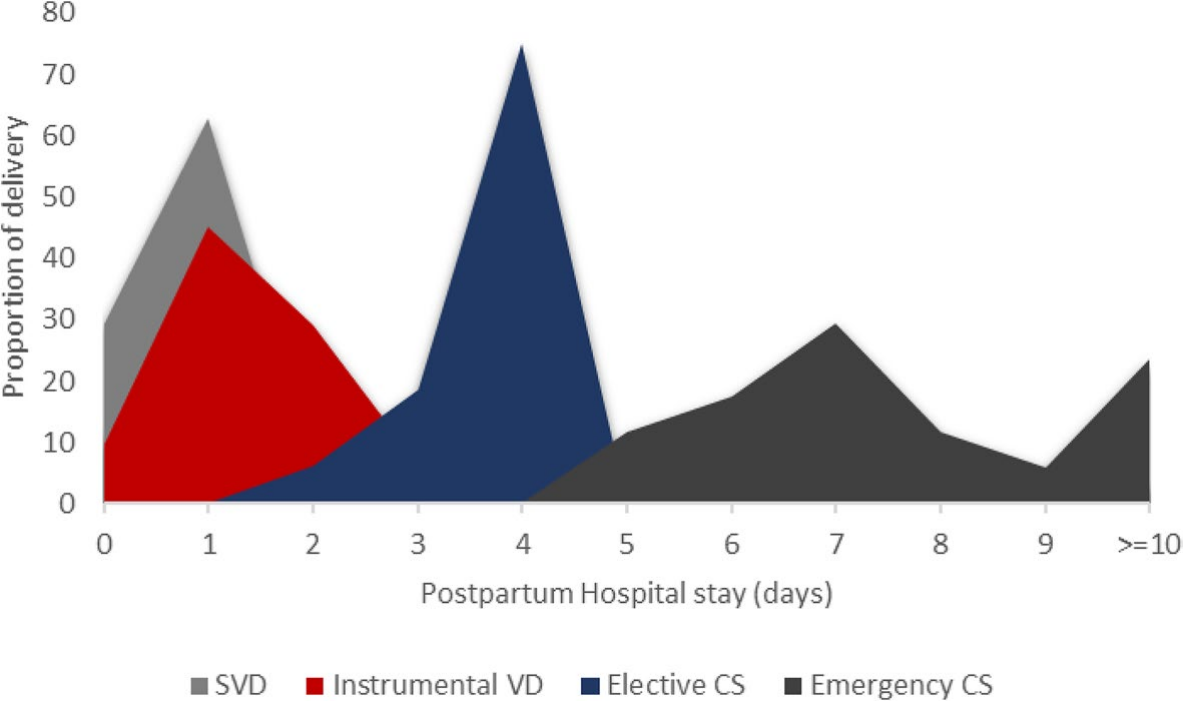
Comparison of the proportion of mothers between modes of delivery and length of hospital stay

A total of 2025 mothers with a median age of 26 (IQR: 22–30) years were subjected to analysis. The median LOS was 1 (IQR: 0–1) day for VD and 6 (IQR: 4–8) days for CD, respectively. Regarding VD, 29% (95% CI: 27–31) of mothers had inadequate stays. Similarly, 32% (95% CI: 18.6-45.3%) had inadequate stays following CD. Figure 2 compares the mode of delivery and length of stay following childbirth. Emergency CD resulted in a higher LOS as compared to other modes of delivery, whereas the majority of elective CD stayed four days. Further, instrumental VD resulted in a higher proportion of LOS > 1 day as compared to spontaneous VD. Table 1 presents the basic maternal and neonatal characteristics of mothers who delivered at Nakfa Hospital during the study period.

**Table 1:** Characteristics of Study Participants

Variables	Vaginal delivery, n (%)	Cesarean delivery, n (%)	Total, n (%)
Age in years			
=<25	905 (46.5)	25 (51)	930 (46.5)
26–32	861 (44.2)	13 (26.5)	874 (43.7)
> 32	181 (9.3)	11 (22.4)	192 (9.8)
Parity			
0	513 (26.7)	31 (62)	544 (27.6)
1–3	905 (47.2)	12 (24)	917 (46.5)
> 3	500 (26.1)	7 (14)	507 (25.7)
Distance from health facility			
=<5 km	1339 (68.2)	21 (42.9 )	1360 (67.5)
> 5 km	624 (31.8)	28 (57.1)	652 (32.5)
Maternal Complication			
Yes	31 (1.6)	11 (22)	42 (2)
No	1944 (98.4)	39 (78)	1983 (98)
Birth weight in kg			
< 2.5	220 (11.3)	5 (10.2)	225 (11.2)
2.5–3.5	1402 (71.8)	33 (67.3)	1435 (71.7)
> 3.5	330 (16.9)	11 (22.4)	341 (17)
Apgar score			
> 6	1882 (96.7)	41 (82)	1923 (96.2)
4–6	11 (0.6)	1 (2)	12 (0.6)
< 4	54 (2.8)	8 (16)	62 (3.2)
Duration stay in days, Median (IQR)	1 (0–1)	6 (4–8)	1 (0–1)

*Abbreviations*: *LOS *Length of stay, *IQR *Interquartile range

### Maternal and neonatal characteristics of CD

In this analysis, the median age of mothers who delivered via CD was 25 (IQR: 20–28),while the majority (51%) were < 25 years old. Moreover, 77.6% of CDs were emergency operations. The majority (80%) of mothers had primary CD. Intraoperative complications were evident in 22% of CDs. Concerning time of delivery, 69.4% of CDs were performed in the hour interval of 8:00–15:00. Regarding neonates, the median birth weight was 3 (IQR: 2.8–3.4) kg, and the majority (67.3%) were between 2.5 and 3.5 kg. The median 5-minute Apgar score was 9 (IQR: 8–10) while the 5-minute Apgar core was > 6 in 82%. Moreover; 9/55 (16.4%) mothers had stillbirths. Notably, 57.1% of mothers traveled greater than 5 km to deliver in Nakfa Hospital (See Table 2 for details).

### Factors associated with LOS following cesarean childbirth

LOS > 4 days following childbirth were significantly associated with emergency CD as compared to elective CD (90.6% Vs 9.4% respectively, *p*- value < 0.001) although the small sample size limits conclusive evidence. In addition, mothers that had emergency CD had significantly higher median LOS following childbirth as compared to elective CD (3, IQR: 3–4 Vs 6, IQR: 5–7; *p*-value = 0.01, Mann-Whitney U test). There was no significant association between other factors and LOS in CD mothers, Table 2.

**Table 2:** Factors associated with LOS following CD

Variables	Population, n (%)	LOS ≤ 4 days, n (%)	LOS > 4 days, n (%)	*P*-value(b)
Age in years, median (IQR)	25 (20–28)	24 (20–28)	25 (20-29.5)	0.6^a^
< 25	25 (51)	8 (53.3)	17 (50)	0.9 (0.08)
25–30	13 (26.5)	4 (26.7)	9 (26.5)	
> 30	11 (22.4)	3 (20)	8 (23.5)	
Parity, median (IQR)	1 (1–2)	2 (1–3)	1 (1–3)	0.7^a^
None	31 (62)	9 (56.3)	22 (64.7)	0.7 (0.6)
< 3	12 (24)	5 (31.30	7 (20.6)	
3 or >	7 (14)	2 (12.5)	5 (14.7)	
Distance from Health facility, median (IQR)	10 (3–33)	6 (3–30)	10 (3–33)	0.4^a^
< 5 km	21 (42.9)	8 (50)	13 (39.4)	0.4 (0.4)
> 5 km	28 (57.1)	8 (50)	20 (60.4)	
Prior CD				
Yes	10 (20)	3 (18.8)	7 (20.6)	0.8 (0.02)
No	40 (80)	13 (81.3)	27 (79.4)	
Type of CD				
Emergency	38 (77.6)	8 (50)	30 (90.6)	**< 0.001 (10.3)**
Elective	11 (22.4)	8 (50)	3 (9.1)	
Intraoperative complications				
Yes	11 (22)	3 (18.8)	8 (23.5)	0.7 (0.1)
No	39 (78)	13 (81.3)	26 (76.5)	
Delivery time				
8:00–15:00	34 (69.4)	13 (81.3)	21 (63.6)	0.3 (2)
16:00–22:00	7 (14.3)	2 (12.5)	5 (15.2)	
23:00–7:00	8 (16.3)	1 (6.3)	7 (21.2)	
Birth weight in kg, median (IQR)	3 (2.8–3.4)	3 (2.5–3.8)	3 (2.8–3.3)	0.6^a^
< 2.5	5 (10.2)	3 (18.8)	2 (6.1)	0.1 (3.5)
2.5–3.5	33 (67.3)	8 (50)	25 (75.8)	
> 3.5	11 (22.4)	5 (31.3)	6 (18.2)	
5 min Apgar score				
> 6	41 (82)	14 (87.5)	27 (79.4)	0.6 (0.7)
4–6	1 (2)	0	1 (2.9)	
< 4	8 (16)	2 (12.5)	2(17.7)	

*Abbreviations*: *LOS L*ength of stay, *IQR *Interquartile range, and *CD *Cesarean delivery. Superscripts: a- Mann-Whitney U test, b- Fisher’s exact test

### Indications for cesarean section

**Fig. 3:**
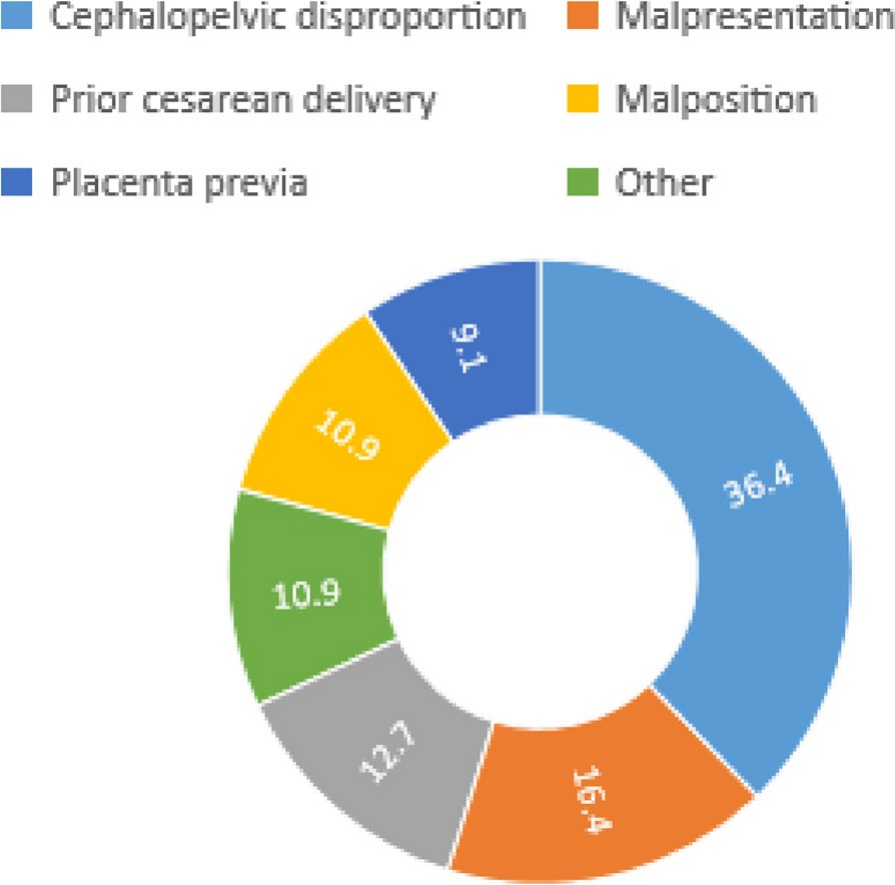
Indication for Cesarean Section

Figure 3 represents indications for CD during the study period. Cephalo-pelvic disproportion accounted for the majority (36.4%) of CD, followed by abnormal presentation of the fetus, prior history of CD, and placenta Previa.

### Maternal and neonatal characteristics for VD

A total of 1975 mothers delivered vaginally from 2020 to 2022. Proportional distribution across categories of age < 26 and 26–32 (46.5% and 44.2% respectively) was observed, whereas only 9.3% accounted for age groups > 32 years. Only 1.6% of the total had maternal complications during childbirth. A history of miscarriage was found in 4.8% of participants. Regarding the time of delivery, a similar distribution was observed in the delivery years 2020, 2021, and 2022 (35.7%, 34.3%, and 29.9%, respectively). Meanwhile, in contrast to CD majority of mothers delivered during the 23:00–7:00 h interval, followed by 8:00–15:00 (44% and 31.3% respectively). Concerning neonates, the majority had birth weights of 2.5–3.5 kg and a 5-minute Apgar score > 6 (71.8% and 96.7%). Stillbirth occurred in 50/1975 (2.5%) of VDs. Furthermore, 0.9% of the total VD mothers were attended by physicians, and the rest were attended by midwife nurses and axillary nurses (49.3% and 49.8% respectively). (See Table 3 for details).

### Factors associated with LOS following vaginal childbirth

**Fig. 4:**
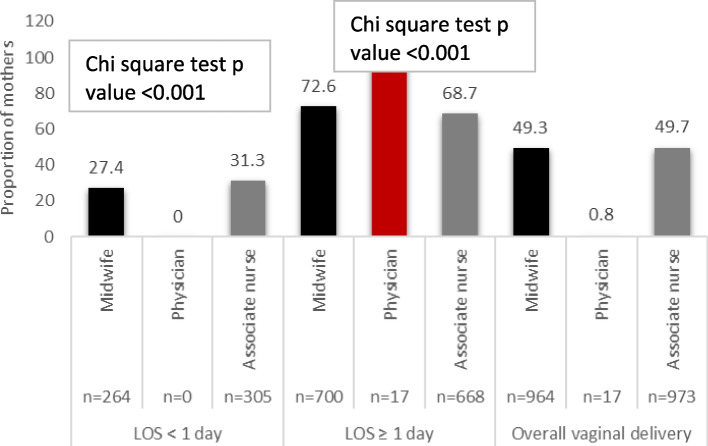
Association between LOS and level of birth attendant

Table 3 presents factors associated with LOS following VD. In this analysis, the presence of maternal complications was associated with LOS > 2 days, with such mothers accounting for 12.5% of LOS > 2 days as compared to only 1.7% and 0.5% of adequate LOS and inadequate stay, respectively, *P*-value < 0.001. Furthermore, Mothers who delivered in the year 2022 accounted for a higher proportion of inadequate LOS as compared to 2021 and 2020 (43.8% vs. 31.1% and 25.1%, respectively, *p*-value < 0.001). Moreover, mothers that delivered in the hour interval 23:00–7:00 accounted for a significantly higher proportion of inadequate LOS (56.1%) as compared to the 8:00–15:00 (29.1%) and 16:00–22:00 h intervals (14.9%), *p*-value < 0.001. Figure 4 compares the proportion of inadequate stays according to the level of the birth attendant. In this analysis, VDs that were attended by physicians had a 0% inadequate stay, whereas 27.4% of deliveries attended by midwives and 31.3% by Associate nurse staff resulted in an inadequate stay (*P*-value < 0.001).

**Table 3:** Factors associated with LOS following VD

Variables	Population, n (%)	LOS < 1 day	LOS 1–2 days, n(%)	LOS > 2 days, n (%)	*p*-value(χ2)
Age in years					
=<25	905 (46.5)	247 (44)	642 (47.7)	16 (41)	0.4 (3.9)
26–32	861 (44.2)	260 (46.3)	584 (43.4)	17 (43.6)	
> 32	181 (9.3)	54 (9.6)	121 (9)	6 (15.4)	
Parity					
0	513 (26.7)	152 (27.1)	352 (26.7)	9 (22.5)	0.7 (2.1)
1–3	905 (47.2)	274 (48.8)	612 (46.5)	19 (47.5)	
> 3	500 (26.1)	135 (24.1)	353 (26.8)	12 (30)	
Miscarriage					
Yes	94 (4.8)	35 (6.1)	53 (4.3)	6 (3.6)	0.1 (3.3)
No	1878 (95.2)	538 (93.9)	1178 (95.7)	162 (96.4)	
Distance from health facility					
=<5 km	1339 (68.2)	378 (66.7)	937 (69.1)	24 (60)	0.3 (2.3)
> 5 km	624 (31.8)	189 (33.3)	419 (30.9)	16 (40)	
Number of birth					
Singleton	1955 (99)	568 (99.3)	1348 (99)	39 (97.5)	0.4 (1.6)
Twin	19 (1)	4 (0.7)	14 (1)	1 (2.5)	
Presentation					
Cephalic	1941 (98.4)	564 (98.6)	1338 (98.3)	39 (97.5)	0.8 (0.4)
breech	32 (1.6)	8 (1.4)	23 (1.7)	1 (2.5)	
Maternal complications					
Yes	31 (1.6)	3 (0.5)	23 (1.7)	5 (12.5)	**< 0.001 (54.3)**
No	1944 (98.4)	570 (99.5)	1339 (98.3)	35 (78.5)	
Mode of delivery					
Spontaneous	1964 (99.5)	569 (99.6)	1356 (99.6)	39 (97.5)	0.3 (2.2)
Assisted	9 (0.5)	2 (0.40	6 (0.4)	1 (2.5)	
Year of Delivery					
2020	706 (35.7)	144 (25.1)	541 (39.7)	21 (52.5)	**< 0.001 (85.7)**
2021	678 (34.3)	178 (31.1)	484 (35.5)	16 (40)	
2022	591 (29.9)	251 (43.8)	337 (24.7)	3 (7.5)	
Delivery time					
Day shift	576 (31.3)	156 (29.1)	407 (32)	13 (38.2)	**< 0.001 (58.7)**
Evening shift	457 (24.8)	80 (14.9)	371 (29.2)	6 (17.6)	
Night shift	810 (44)	301 (56.1)	494 (38.8)	15 (44.1)	
Newborn gender					
Male	968 (49.5)	273 (48.4)	673 (49.8)	22 (55)	0.6 (0.8)
Female	987 (50.5)	291 (51.6)	678 (50.2)	18 (45)	
Birth weight					
< 2.5	220 (11.3)	74 (13.1)	138 (10.2)	9 (22.5)	0.058 (8.8)
2.5–3.5	1402 (71.8)	383 (67.5)	993 (73.8)	26 (65)	
> 3.5	330 (16.9)	110 (19.4)	215 (16)	5 (12.5)	
Apgar score					
> 6	1882 (96.7)	555 (97.7)	1290 (96.3)	37 (92.2)	0.1 (6.2)
4–6	11 (0.6)	3 (0.5)	7 (0.5)	1 (2.5)	
< 4	54 (2.8)	10 (1.8)	42 (0.5)	2 (5)	
Degree of birth attendant					
Associate nurse	973 (49.8)	305 (53.6)	641 (47.7)	27 (67.5)	**< 0.001 (37.8)**
Midwife	964 (49.3)	264 (46.4)	690 (51.3)	10 (25)	
Physician	17 (0.9)	0	14 (1)	3 (7.5)	

*Abbreviations*: *LOS *Length of stay, *Km *Kilometers, *Kg *Kilograms, *IQR *Interquartile range and, *VD *(vaginal delivery)

### Hospital LOS following vaginal childbirth stratified by maternal and neonatal characteristics

Table 4 displays the median LOS stratified by maternal and neonatal characteristics. Mothers that had complications during VD had longer median LOS as compared to those without complications (1 (IQR: 0–1 days) Vs 1 (IQR: 0–2 days); *p*-value < 0.001, Mann-Whitney U test). Regarding the time of delivery, the median LOS following VD in the year 2020 was significantly higher relative to 2021 and 2022 (1 (IQR: 1–1) days Vs 1 (IQR: 0–1) days, *p*-value < 0.001; Kruskal Wallis test). Moreover; VD in the hour interval 16:00–22:00 resulted in longer stay as compared to 23:00–7:00 and 8:00–15:00 [1 (IQR: 1–1) days Vs 1 (IQR: 0–1) days, *p* < 0.001; Kruskal Wallis test]. VD attended by a physician had significantly longer median LOS as compared to midwives and Associate nurse staff [1 (IQR: 1-1.25) Vs 1 (IQR: 0–1) days for midwives and Associate nurse staff; *p*-value = 0.002, Kruskal Wallis test]. Concerning neonates, those with 5 min Apgar score less than 4 had significantly higher median LOS of 1 (IQR: 1-1.5) days as compared to 1 (IQR: 0–1) days for a score of > 6; *p*-value = 0.008, Kruskal Wallis test.

**Table 4:** Comparison of LOS following vaginal birth stratified by maternal and neonatal characteristics

Variables	Median LOS (IQR) in days	*p*-value
Age in years		
< 26	1 (0–1)	0.77^b^
26–32	1 (0–1)	
> 32	1 (0–1)	
Parity		
0	1 (0–1)	0.25^b^
1–3	1 (0–1)	
> 3	1 (0–1)	
Miscarriage		
No	1 (0–1)	0.07^a^
Yes	1 (0–1)	
Distance from health facility in Km		
=<5	1 (0–1)	0.83^a^
> 5	1 (0–1)	
Number of fetus		
Singleton	1 (0–1)	0.21^a^
Twin	1 (1–1)	
Fetal Presentation		
Cephalic	1 (0–1)	0.42^a^
breech	1 (0–1)	
Maternal complications		
No	1 (0–1)	**< 0.001**^**a**^
Yes	1 (0–2)	
Mode of delivery		
Spontaneous VD	1 (0–1)	0.52^a^
Instrumental VD	1 (0–2)	
Year of Delivery		
2022	1 (0–1)	**< 0.001**^**b**^
2021	1 (0–1)	
2020	1 (1–1)	
Delivery time		
8:00–15:00	1 (0–1)	**< 0.001**^**b**^
16:00–22:00	1 (1–1)	
23:00–7:00	1 (0–1)	
Birth weight in kg		
< 2.5	1 (0–1)	0.056^b^
2.5–3.5	1 (0–1)	
> 3.5	1 (0–1)	
5 min Apgar score		
> 6	1 (0–1)	**0.008**^**b**^
4–6	1 (0–1)	
< 4	1 (1-1.5)	
Highest level of birth attendant		
Physician	1 (1-1.25)	**0.002**^**b**^
Midwife	1 (0–1)	
Associate nurse	1 (0–1)	

*Abbreviations*: *LOS *Length of stay, *Km *Kilometers, *Kg *Kilograms, *IQR *Interquartile range and *VD *Vaginal delivery

Superscripts: a- Mann-Whitney U test; b- Kruskal Wallis test

### Multivariate analysis for determinants of LOS following vaginal delivery

Table 5 shows the results of the negative binomial regression. In this analysis, the presence of maternal complications was associated with a longer stay (Adjusted IRR = 2.8, 95% CI: 1.6-5, *p*-value < 0.001). In addition, as compared to 2022, the delivery years 2020 and 2021 were associated with longer LOS following childbirth (Adjusted IRR = 1.5, 95% CI: 1.2–1.8, *p*-value < 0.001 and IRR = 1.4 95% CI: 1.2–1.7, *p*-value < 0.001 respectively). Furthermore; delivery time was also found to be associated with LOS following childbirth. Mothers that delivered in the time interval 23:00–7:00 were likely to stay shorter as compared to those that delivered in the interval 8:00–15:00 (Adjusted IRR = 0.8, 95% CI: 0.7–0.9, *p*-value = 0.03).

**Table 5:** Negative binomial regression: factors associated with LOS following vaginal birth

Variables	Crude Incidence rate ratio (95% CI)	*p*-value	Adjusted Incidence rate ratio (95% CI)	*p*-value
Age in years				
< 26	*1 (Ref)*			
26–32	1.03 (0.8–1.3)	0.7		
> 32	1.03 (0.8–1.1)	0.6		
Parity				
0	*1 (Ref)*			
1–3	0.87 (0.7–1.04)	0.1		
> 3	0.88 (0.71–1.04)	0.1		
Miscarriage				
No	*1 (Ref)*			
Yes	1.05 (0.7–1.4)	0.7		
Distance from health facility (km)				
=<5	*1 (Ref)*			
>5	0.9 (0.8–1.1)	0.9		
Number of fetus				
Singleton	*1 (Ref)*			
Twin	1.1 (0.6–2.2)	0.5		
Fetal Presentation				
Cephalic	*1 (Ref)*			
breech	1.08 (0.6–1.7)	0.7		
Maternal complications				
No	*1 (Ref)*		*1 (Ref)*	**< 0.001**
Yes	2.6 (1.7-4)	< 0.001	2.8 (1.6-5)	
Mode of delivery				
Spontaneous VD	*1 (Ref)*			
Instrumental VD	1.4 (0.6–3.5)	0.4		
Year of Delivery				
2022	*1 (Ref)*		*1 (Ref)*	
2021	1.4 (1.2–1.70)	**< 0.001**	1.4 (1.2–1.7)	**< 0.001**
2020	1.5 (1.3–1.8)	**< 0.001**	1.5 (1.2–1.8)	**< 0.001**
Delivery time				
8:00–15:00	*1 (Ref)*		*1 (Ref)*	
16:00–22:00	1.05 (0.8–1.2)	0.5	1.1 (0.9–1.3)	0.3
23:00–7:00	0.8 (0.7–0.9)	**0.02**	0.8 (0.7–0.9)	**0.03**
Birth weight in kg				
< 2.5	*1 (Ref)*			
2.5–3.5	0.9 (0.7–1.1)	0.4		
> 3.5	0.8 (0.6-1)	0.1		
5 min Apgar score				
> 6	*1 (Ref)*			
4–6	1.2 (0.5–2.7)	0.6		
< 4	1.3 (0.9–1.9)	0.1		
Highest level of birth attendant				
Physician	*1 (Ref)*			
Midwife	0.5 (0.21–0.98)	0.03		
Associate nurse	0.53 (0.29–0.99)	0.04		

*Abbreviations*: *CI *Confidence interval, *km *kilometers, *kg *kilograms, *LOS* Length of stay and *VD *Vaginal delivery

## Discussion

Childbirth is a physiologic process considered spontaneous and safe when it takes place in health facilities, but complications can arise regardless of the place of delivery. Therefore, it’s important to for mothers to stay in health facilities where there is a skilled birth attendant who can identify and respond to any complications. The WHO also advocates for an adequate postpartum stay for the timely detection of maternal and neonatal complications.

In this study, nearly one-third of mothers had inadequate LOS following vaginal birth. The magnitude of inadequate LOS remains a challenge in SSA, with a substantial proportion of mothers staying in health facilities for less than 1 day, Tanzania (65.7%) [6], Ethiopia (65.2%) [12], Ghana (35%), and Swaziland (18.5%) [9]. More importantly, a study evaluating the provision of postpartum care in 33 SSA countries noted substantial variation between countries, with the median country percentage being 71.7% and ranging from 26.6% in Swaziland to 94.4% in Burkina Faso. Nonetheless, the investigators reported that less than 50% of mothers reported the provision of pre-discharge check-ups following childbirth in nearly one-fourth of the SSA countries included in the study [26].

In Africa, discharge within 24 h following vaginal facility delivery is considered an institutional norm and a societal expectation, except in the presence of a complication [6]. A study in Tanzania found that although the time of discharge was decided by healthcare providers, women were happy to leave before 24 h, either due to a longing to be home or due to some facility-related discontentment [6]. Such factors may perhaps be attributable to this setting as well, since Nakfa Hospital is a remote health facility with limited infrastructure. No doubt, inadequate LOS can have detrimental effects on both maternal and neonatal health [19, 20]. Appropriate postpartum LOS provides enough time for the assessment of the mother and newborn. It also provides us with critical opportunities for counseling about exclusive breastfeeding and to discuss the mother’s childbirth experience during their hospital stay. Therefore; efforts to improve adherence to the WHO recommendation on the minimum length of stay after childbirth, in addition to improving the facility environment to enable a longer stay, should be prioritized in this setting.

Unsurprisingly, CD resulted in a longer median LOS as compared to SVD in this population. Reports generally suggest a longer LOS following CD [21, 26]. Women who have caesarean deliveries, irrespective of demographic and obstetric characteristics, have a higher risk of maternal morbidity compared to VDs [27, 28], hence a longer LOS. Furthermore, the median LOS was 6 days in this setting. Prior Reports have documented that LOS following CD has large variability (2.5–9.3 days) across different contexts [10]. Lower median LOS following CD has been reported in Sudan [29], Australia [30], and the US [25, 31]. On the other hand, comparable LOS in mothers who delivered via CD has been reported in India [21], Malawi, and Ghana [9]. When compared with elective CD, Emergency CD was associated with a longer LOS. A prior study in Sudan reported a relatable finding [25]. This can perhaps be explained by the fact that emergency CD is associated with more maternal complications and, hence, longer hospitalization [31]. Thus, early detection of high-risk mothers through the provision of high-quality antenatal care and follow-up should be worked upon.

Interestingly, deliveries attended by a physician were unlikely to result in an inadequate stay as compared to midwives and Associate nurse staff. This finding concurs with prior literature. A study in Nepal concluded similar findings: delivery attended by an Associate nurse staff was associated with a shorter length of stay, as compared with delivery attended by a physician and a nurse, even after adjusting for complications and other factors (IRR = 0.86, 95% CI 0.75 to 0.98) [32]. No doubt, equipped with a higher extent of academic and clinical training, doctors would have better rates of adherence to the standard LOS and a superior capacity for identifying both maternal and neonatal complications. Indeed; a study evaluating the competence of healthcare professionals in diagnosing and managing obstetric complications identified that median scores differed significantly between different levels of health professionals, 11 (IQR: 7–14) points for medical doctors vs. 8 (IQR: 6–10) points for nurses and midwives (*p* = 0.0002) [33]. More importantly, suboptimal provision of postpartum care for women giving birth in health facilities in 33 sub-Saharan African countries was found to be associated with the level of health professionals. According to this report, as compared to physicians, midwives and skilled birth attendants had a lower provision of postpartum check-ups (AOR = 0.74, 95% CI: 0.69–0.78 and 0.14, 95% CI: 0.12–0.15, respectively) [26]. All things considered, this factor may capture larger issues of insufficient staffing, poor training, and low adherence to guidelines in this setting. Moreover; Nurses or midwives usually attend less risky births and consult on complicated births with physicians that require an indicated extension of stay. Another explanation is that dimensions related to the health system and women or family dimensions that might be drivers for inappropriately short stays are more likely to have an effect on these less risky childbirths.

The present study found maternal complications were an independent determinant of longer LOS following VD. The finding is consistent with Prior literature reports [29, 34]. The provision of post-partum care is essential for mothers following childbirth, even more so for mothers who experience complications during labor. Therefore, it’s not surprising for mothers who incur complications to stay longer in a hospital where postnatal care is available. Indeed, length of stay has been observed to improve the utilization of postpartum care [21]. Moreover, mothers who delivered neonates with a 5-minute Apgar score less than 4 had a significantly longer median LOS. The results of our study collaborate with existing evidence. Indeed, previous investigators have noted that neonatal complications result in a longer duration of stay [29]. The Apgar score is a simple and replicable method to quickly and summarily assess the health of newborn children immediately after birth [35]. Generally, the Apgar score is an indicator of how well the neonate adapts to the transition from life in utero to the environment. No wonder neonates with lower 5-minute Apgar scores need better postnatal care, for example, admission to the neonatal ICU, which requires mothers to stay an additional day to breastfeed their babies.

Concerning time of delivery, both delivery hour and delivery year were determinants of LOS. On the latter, mothers who delivered in the years 2020 and 2021 had higher LOS as compared to 2022. The impact of COVID-19 on healthcare systems can perhaps be invoked to explain the observed difference in LOS. Fragile health systems in developing countries faced organizational and resource constraints when confronted with the Covid 19 pandemic. A systematic review of 81 studies across 20 countries reported a median 37% reduction in overall healthcare utilization and a median 42% reduction in health facility visits [36]. In contrast, in this study, the proportion of VD remained fairly stable throughout the study period [706 (35.7%) in 2020, 678 (34.3%) in 2021, and 591 (29.9%) in 2022]. Despite the stable proportion of VD across the years, mothers that delivered in the years 2020 and 2021 had longer median LOS and higher incidence rate ratios of LOS. In Eritrea, lockdown was instituted in early 2020 and lasted all the way to the latter half of 2021. It is likely that a lack of transportation coupled with poor accessibility infrastructure resulted in postpartum mothers staying in the facility longer, even more so during the lockdown of 2020 and 2021. Moreover, this study uncovered that mothers who delivered between 22:00 and 8:00 had a significantly shorter stay as compared to those who delivered between 8:00–21:00. Similarly, Ikeda et al. noted that mothers who gave birth between 11:00 and 02:00 were more likely to stay longer (11:00–19:00: IRR = 1.11, 95%CI 1.01 to 1.22, 20:00–02:00: IRR = 1.15, 95%CI 1.02 to 1.30), as compared with those who gave birth between 03:00 and 10:00 [32]. Nakfa subzone is located in mountainous topography with poor access, road infrastructure, and transportation options. In this setting, going home after dark can be difficult for postpartum mothers given the lack of transportation. After it gets dark, going home with a postnatal mother and child without transportation can be difficult in such settings. In the open literature, evidence suggests such factors can be influential in similar settings [32].

Lastly, assisted VDs were infrequent in this study, with only 9 (0.5%) of VDs. In contrast, in a retrospective review of records involving 4396 mothers from nine sub-Saharan African countries, the proportion of assisted VD was 18% [37]. Similarly, a study conducted in Tanzania reported a higher rate of assisted VD (3.9%) [38]. This low rate of instrumental VD could be attributed to incorrect documentation, as investigators in the above study in Tanzania noticed that instrumental VD were sometimes recorded by midwives as spontaneous VDs. Another reason is that healthcare professionals in resource-limited settings are often less experienced with instrumental VD techniques [37].

### Limitations and strengths of the study

This study is valuable in that it is based on every mother’s details throughout the study period. Moreover; the large sample size, particularly for VD, enables you to compare different variables with significant magnitude. Despite this, the study is not without limitations. The small sample size on CD in the hospital precludes conclusions concerning factors associated with cesarean delivery. The retrospective nature of the study, resulting in missing covariate data, can also be listed as a drawback. Of note, 8% of mothers were excluded due to key missing data. Including critical socioeconomic data on mothers would have given the study a broader scope. Further, the study didn’t explore perspectives from health providers or information about patient satisfaction. Moreover; factors that might influence women’s decisions for facility delivery or expectations on LOS, such as community efforts, were not evaluated. 

## Conclusion

The study, the first of its kind in Eritrea, provides results on a range of maternal LOS-related issues. In this study, a substantial proportion of mothers stayed too short post VD to allow adequate postnatal care. Moreover; Delivery years 2020 and 2021 were associated with longer LOS following VD. In contrast, deliveries attended by midwives and associate nurse staff, and deliveries in the hour interval 23:00–7:00 were associated with shorter stays. Going forward, more emphasis should be placed on enforcing guidelines on LOS in health facilities, and on the provision of antenatal care for the early diagnosis of maternal complications in pregnancy. In addition, further research into the determinants of LOS following CD and a survey to assess the knowledge and understanding of hospital staff should be done in the future.

## Data Availability

The dataset supporting the conclusions of this article is available from the corresponding author on reasonable request.

## Abbreviations

CD: Cesarean delivery
CI: Confidence interval
LOS: Length of stay
IQR: Interquartile range
IRR: Incidence rate ratio
MCU: Maternity Care Unit
MOH: Ministry of Health
SSA: Sub-Saharan Africa
UN: United Nations
VD: Vaginal Delivery
WHO: World Health Organization
